# Considerations for Establishing Peptide Receptor Radionuclide Therapy: A Nationally Coordinated, Collaborative, and Equitable Service for New Zealand

**DOI:** 10.1055/s-0045-1807726

**Published:** 2025-05-01

**Authors:** Rachelle Steyn, Andrew Henderson, Karin Wells, Trish Mead, Yasmin Rennie, Avril Hull, Liana Meredith, Michelle Sullivan, Saskia Simmers, Jenny Davidson, Tame Hauraki, Dragan Damianovich, Veronica Boyle, Ben Lawrence

**Affiliations:** 1 Department of Nuclear Medicine, Auckland City Hospital, Grafton, Auckland, New Zealand; 2 Mercy Radiology, Epsom, Auckland, New Zealand; 3 Department of Medical Oncology, Auckland City Hospital, Grafton, Auckland, New Zealand; 4 Neuroendocrine Cancer New Zealand, Meadowbank, Auckland, New Zealand; 5 Department of Oncology, School of Medical Sciences, Faculty of Medical and Health Science, University of Auckland, Auckland, New Zealand; 6 School of Medicine, Faculty of Medical and Health Science, University of Auckland, Auckland, New Zealand

**Keywords:** New Zealand, NET, PRRT, implementation, multidisciplinary, health equity

## Abstract

Inspired by international frameworks, New Zealand established a nationally coordinated peptide receptor radionuclide therapy service. This article reflects on the key steps involved in building the service, including the formation of a national neuroendocrine tumor (NET) multidisciplinary meeting, the role of patient advocacy, and the integration of local research. The successful creation of the service, despite significant challenges, demonstrates the value of collaboration between clinicians, government, universities, and patient groups in achieving equitable, high-quality care.

## Background

New Zealand (NZ) was ranked 14th in per capita health spend in the Organisation for Economic Co-operation and Development in 2022 1 and provides publicly funded health care at no cost to residents. 2 A comprehensive neuroendocrine tumor (NET) service requires specialized multidisciplinary expertise, which is difficult to marshal in a small country with a population of 5 million. Inspired by the European Neuroendocrine Tumour Society (ENETS) Centres of Excellence framework 3 and evidence from the NETTER-1 trial, 4 NZ used a collaborative approach to establish a peptide receptor radionuclide therapy (PRRT) service comparable to international gold standard.

**Fig. 1:**
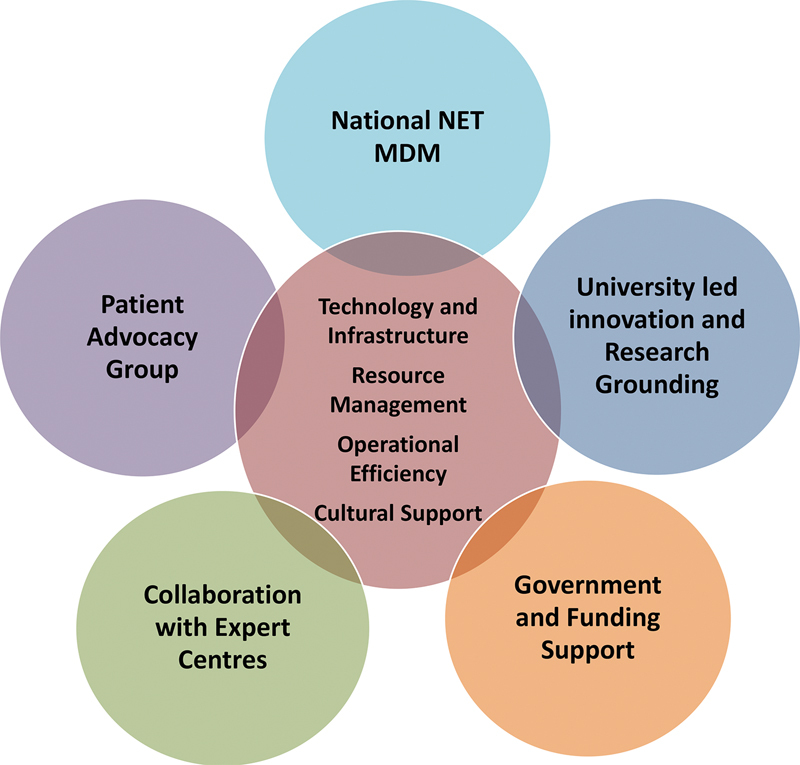
Key success factors that contributed to the initiation, establishment, and maturation of New Zealand's peptide receptor radionuclide therapy (PRRT) service.

This article outlines the key elements that contributed to the initiation, establishment, and maturation of NZ's nationally coordinated PRRT service ( Fig. 1 ). Some elements were recognized in the prospective planning process, but we augment this with the easy wisdom of hindsight, to assist groups considering the establishment of a PRRT service.

## Begin with a National NET Multidisciplinary Meeting

NETs are uncommon, heterogeneous tumors requiring complex individualized management strategies. Treatment planning for people with NETs in multidisciplinary meetings (MDMs; also known as tumor boards) is evidence-based, improves outcomes, and can lead to altered management in up to 61% of cases. 5 NET MDMs are therefore the foundation for clinical care including selection of patients for PRRT.

The NZ NET MDM was designed at national clinical meetings in 2012 and 2014. The meetings were led by a small team of medical specialists and scientists who began the “NETwork!” research project, which combined NET outcome measurement, epidemiology, tissue collection, genomics, and clinical care. The academic component provided energy, partial salary support for a clinical lead, rigor in planning, and attracted like-minded clinicians working together to understand NETs.

Initially, MDMs in four to six sites were planned across the country. Due to limited administrative and funding support, a single NET MDM was established in one center, which gradually opened to national referrals, ensuring that any clinician/patient in NZ has access to expert multidisciplinary advice.

The structure has since evolved to a core group of regularly attending specialists in nuclear medicine (NM), oncology, endocrinology, pathology, radiology, surgery, and NET clinical nursing. There is an ongoing process to recruit other specialists including gastroenterologists, cardiologists, radiation oncologists, and dieticians to regularly attend; currently, these specialists are individually invited to attend if a case requires their input.To evaluate the impact that the NET MDM had on clinical management an internal retrospective audit of the first 103 cases was performed. Three attending specialists retrospectively and independently compared patients' medical records to MDM recommendations. The results showed significant clinical impact: diagnosis changed in 17%, the World Health Organization grading in 14%, and radiological staging in 25%. Patient management changed in 50%, avoiding surgery in 9%, chemotherapy in 3%, and shifting to observation in 8%. MDM recommendations were followed in 74% of cases.

Subsequently, the frequency of meetings increased from monthly to weekly, with funding secured for a coordinator (2017), online pro forma (2018), and Zoom video platform (2019). The online format allowed expert clinicians from multiple centers to join the MDM team. The national NET MDM unified NZ NET specialists. Increasing case volume enhanced experience and improved the quality of recommendations. The meeting is currently the sole point for PRRT referral.

## Connect with a Strong Patient Advocacy Group

Neuroendocrine Cancer New Zealand (NECNZ) was founded in 2013 (initially as the Unicorn Foundation NZ) after advocates attended the first national clinical NET meeting in 2012, to address increasing demand for advocacy, support, access to treatment, and education. Its growth paralleled the expansion of NET clinical treatment teams. NECNZ was instrumental in securing local access to gallium-68 (Ga-68) DOTATATE (dotatate) positron emission tomography/computed tomography (PET/CT), supporting NET research and establishing the PRRT service through awareness campaigns, patient support, and collaboration with health care institutions.

## Use Local Research to Define the Size of the Problem

Epidemiological data from the NETwork! research group indicated that the age-adjusted incidence rate of NET cases in NZ increased from 5.7 per 100,000 in 2008 to 6.2 per 100,000 in 2012. 6 This rate is comparable to findings from the United States, where 6.98 per 100,000 patients were diagnosed with NET in 2012. 7 Among patients who underwent radiological staging at diagnosis or within 12 months, 46% were found to have either lymph node involvement or distant metastases. Using the number of patients with metastatic disease, and estimates of somatostatin receptor expression from the international literature, we estimated that 30 people with NETs would be eligible for PRRT each year. This estimate served as the foundation for the subsequent PRRT business case.

## Establish Provision of Ga-68 DOTATATE PET/CT

**Fig. 2:**
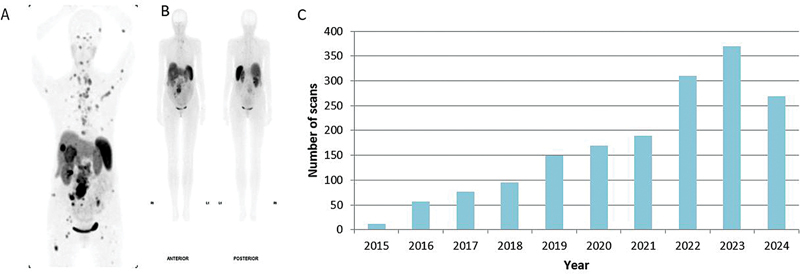
The first dotatate positron emission tomography/computed tomography (PET/CT) performed in New Zealand (NZ) ( **A** ) compared with Tekrotyd ( **B** ). The improved quality of dotatate PET/CT ( **A** ) compared with Tekrotyd ( **B** ) demonstrated diagnostic superiority to health funders resulting in increased local access ( **C** ).

Before 2015, somatostatin receptor imaging was conducted using Tekrotyd. 8 NZ does not have publicly provided PET cameras and PET/CT work is contracted to commercial providers, none who were able to provide Ga-68 imaging. Patients travelled to Australia for Ga-68 DOTATATE (dotatate) PET/CTs at their own personal expense resulting in significant inequity. NECNZ launched a fundraising campaign to support purchase of a Ga-68 generator by a commercial radiology provider. The first dotatate PET/CT scan in NZ was performed in November 2015 ( Fig. 2 ). Access to Ga-68 radiopharmaceuticals has subsequently expanded as further staff received appropriate training and supervision in good manufacturing practice (GMP) compliant synthesis. Access to publicly funded PET was strictly managed through the National NET MDM. Initially, dotatate scans were capped at 50 patients nationally per year and limited to presurgical planning, but criteria later expanded to assess suitability for PRRT ( Fig. 2C ).

## Establish Local Synthesis of Lutetium-177 DOTATATE

The importation of Lutetium-177 (Lu-177) is facilitated by Global Medical Solutions, Auckland, NZ. Radiopharmaceutical synthesis followed GMP-compliant protocols using high-purity reagents and the Modular-Laboratory Pharm Tracer (Eickert and Ziegler) system. Local GMP production of Lu177-DOTATATE improved cost-efficiency, reduced clinical site workloads, and provided high-quality products with extended shelf lives, opening the possibility to supply other centers in NZ as the service grows. The production and use of Ga-68 and Lu-177 DOTATATE in NZ adhere to the Ministry of Health's Office of Radiation Safety C2 Code of Practice 9 and the Radiation Safety Act (2016). 10 Regulatory compliance and collaboration were essential in establishing a safe and effective PRRT service.

## Develop a Shared Understanding of the Problem with Regulators

Defining gold standard NET care is complex for clinicians and extremely difficult for nonclinical regulators who need to consider and compare multiple applications for health care funding. The NETTER-1 trial published in 2016 4 provided randomized control evidence of efficacy of PRRT. NECNZ initiated advocacy for PRRT in 2017, and clinicians worked with government organizations to help them to understand the place of PRRT in the treatment of people with NETs by taking the unusual step of submitting a document that described NETs and NET care to the NZ pharmaceutical funding agency PHARMAC, prior to making a submission for PRRT funding. The initial approach allowed PHARMAC to understand that not all people with NETs need or are suitable PRRT, thus reducing the potential financial exposure for a drug funding agency.

## Create Evidence-Based Funding Criteria

To optimize clinical outcomes and ensure sustainable health care investment, NET specialists and PHARMAC established funding criteria based on the efficacy data from the NETTER-1 trial 4 but expanded beyond the midgut primary site based on retrospective series showing similar efficacy provided somatostatin receptor expression was present. 11 PRRT funding eligibility required a recommendation from the National NET MDM and was restricted to patients with advanced, inoperable, progressive, well-differentiated NETs with somatostatin receptor-expressing disease. For patients meeting the funding criteria the cost per PRRT infusion treatment cycle was estimated to be $NZ 8,775 ($US 5,018).

## Partner with Central Cancer Leadership and Never Waste a Crisis

**Fig. 3:**
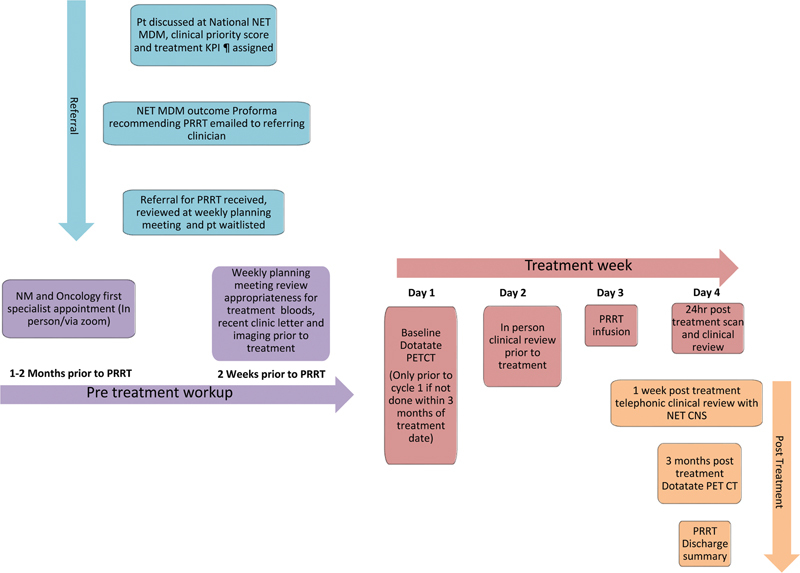
Patient pathway. ¶ KPI, key performance index for treatment delivery date.

In October 2019, funding for accelerated access to funded PRRT in Australia for the most urgent NZ patients was approved as an interim measure while a PRRT service was considered and developed. This arrangement took a critical turn in 2020 when the coronavirus pandemic progressively reduced access to overseas health care. By July 2020 strict border closures completely halted access to PRRT in Australia. An urgent cross-government round table with key leadership from the National Cancer Control Agency, Te Aho o Te Kahu, resulted in expedited funding approval of publicly provided PRRT, with the goal of setting up a national PRRT service within 6 to 8 weeks. This timeframe posed an immense challenge, requiring rapid coordination of resources, infrastructure, and specialist expertise while delivering international practice standards ( Table 1 and Fig. 3 ).

**Table 1:** Approach to setting up the PRRT service in 6 to 8 weeks

Component	Details
Resource consolidation	Consolidation of PRRT services to a single, centralized center at Auckland City Hospital due to limited resources
Infrastructure	Nuclear medicine department: Modified and adapted existing equipment to safely handle, dispense, and administer Lutetium-177 dotatate Treatment rooms: Repurposed two shielded rooms in the medical oncology ward, originally equipped for high-dose I-131 therapy, for PRRT Acute admission space: Created capacity in the medical oncology ward for patients requiring admission after PRRT treatment National Travel Assistance Scheme (NTA): Provided financial assistance for travel and accommodation for patients and support persons from elsewhere in NZ
Regulatory requirements	All activities were made compliant with the Ministry of Health ORS C2: Code of Practice for Nuclear Medicine 9 and the Radiation Safety Act (2016) 10
Lutetium-177 dotatate access	Collaboration with a commercial radiology practice, which had the required equipment and expertise to ensure supply of lutetium-177 dotatate was initiated
Maintaining a multidisciplinary approach	An onsite multidisciplinary treatment team including nuclear medicine, medical oncology, endocrinology, nursing, and medical physics was established
Collaboration with established centers of excellence	Collaboration with expert PRRT treatment centers, notably the Peter MacCallum Cancer Centre (Melbourne) and the Royal North Shore Hospital (Sydney), provided crucial guidance in developing treatment protocols and patient pathways ( Fig. 3 )

Abbreviations: I-131, iodine-131; NZ, New Zealand; ORS, Office of Radiation Safety; PRRT, peptide receptor radionuclide therapy.

## Take Advice from Regional Expert Centers

NZ's closest neighbor Australia has PRRT centers of excellence assessed using the ENETS program. The teams at Peter MacCallum Cancer Centre (Melbourne) and the Royal North Shore Hospital (Sydney) generously shared clinical protocols and provided mentoring. Video-linked MDMs were conducted for difficult cases. This support, based on knowledge developed over decades of PRRT practice, was critical to establish a safe and effective service in NZ.

## Maturation of the Service

Since establishment, the service has been in a constant state of refinement and evolution to ensure robustness and quality of care. Six key areas of improvement were:

1. Consolidating infrastructure, staffing, training, and continuous professional development:

*Infrastructure* : Auckland Hospital was able to meet the national 9 10 and international 12 standards for the safe delivery of radionuclide therapies including radiation protection, storage, and disposal thereof. Dedicated lead lined shielding rooms originally used for administration of high-dose iodine-131 (I-131) were repurposed for administration of Lu-177 dotatate. Initially, only two of the four dedicated lead lined shielding rooms in the hospital were available for use. In July 2022, following the approval of an additional business case, a third room was made available for use to increase patient throughput.

When the service was first established, patients were admitted to the treatment rooms for the night following their PRRT infusion for monitoring of secretory crises or pain flare. As a newly introduced service, for a group of patients who had often been waiting with extensive NET disease, the treating team prioritized patient safety to ensure they could manage any potential adverse events. Over time, as the team gained experience and confidence in the safety profile of PRRT, the treatment protocol evolved. Patients who remained stable postinfusion are now discharged the same day (the vast majority), while those requiring admission are accommodated within the adjacent medical oncology ward. This transition optimizes admission and staffing resources.

A further challenge encountered during the establishment of the service was the lack of a specific protocol for the admission of radioactive patients to the intensive care unit (ICU). Additionally, intensive care teams had not received basic training on how to safely manage radioactive patients in an acute setting. Recognizing the importance of addressing these gaps, significant effort was dedicated to education, training, and collaboration with critical care teams. This involved developing a structured protocol to guide emergency response teams in safely managing radioactive patients on the ward and ensuring a clear framework for the admission of radioactive patients to the ICU. Importantly, this protocol was designed not only for PRRT patients but also for those receiving high-dose metaiodobenzylguanidine (MIBG) therapy, ensuring a standardized approach to the acute management of all radioactive patients requiring intensive care.

*Staffing* : To expand treatment capacity and advance service development and training, an additional NM specialist with expertise in theranostics was recruited in 2022. This specialist is accredited by the Joint Training Committee of the Australasian Colleges of Radiology and Internal Medicine and is a fellow of the Australasian Association of Nuclear Medicine Specialists, meeting Australasian and international criteria for performing theranostics, as well as for training and supervision. 13 Alongside the NM specialist, a medical officer in NM with an MBChB qualification was also recruited to support service delivery. Recruiting medical officers in NM is vital, given the ongoing shortage of theranostics-skilled NM specialists not only in NZ but also globally 14 and in particular developing countries. 15 While the medical officer collaborates closely with the specialist, the overall responsibility for patient care and service development remains with the NM specialist. The onsite theranostics treatment team now consists of three NM specialists, one medical officer, four NM medical imaging technologists (NM MITs), two medical oncologists, one endocrinologist, two NET clinical nurse specialists, and one medical physicist.

*Training* : Auckland City Hospital, a tertiary center, received accreditation for NM training, including theranostics, in 2024 by the joint training committee of the Australasian Colleges of Radiology and Internal Medicine. The facility currently meets the criteria to be classified as a Category 1: Basic clinical Theranostics Training Center. 13

Theranostics has always been within the scope of practice for NM MITs in NZ; however, their role has traditionally been limited, working alongside NM specialists, oncologists, and license holders. With advancements in theranostics, NM MITs now play a significantly larger role, necessitating further skill development in this specialized field. To address this, the University of Auckland has introduced a 12-week theranostics course, covering key areas such as imaging and quantification, dosimetry, theranostics services and roles, the establishment of theranostics services, radiation safety and protection, and clinical applications. This course, launching in July 2025, is fully online and forms a compulsory component of the Nuclear Medicine Postgraduate Diploma in Health Sciences. Additionally, it is available as a Certificate of Proficiency, for registered MITs as an independent course for Continuing Professional Development. Integrating dedicated theranostics training within the NM MITs current scope of practice is essential in this rapidly advancing field. This initiative positions NM MITs to adopt leading practice standards and take on a more comprehensive role in the delivery of theranostic therapies. 16

Course link: https://courseoutline.auckland.ac.nz/dco/course/MEDIMAGE/729/1255

*Ongoing professional development* : Ongoing professional development is essential to ensure a high-quality, evolving PRRT service. To support this, short teaching sessions have been integrated into the weekly PRRT planning meetings, with teaching responsibilities rotating between NM, medical oncology, and endocrinology. This structured approach reinforces proficiency in patient care, medical knowledge, interpersonal skills, practice-based learning, professionalism, and ethical behavior, ensuring a well-rounded, multidisciplinary learning environment.

Beyond internal training, close collaboration with expert centers remains a priority, allowing the team to learn through discussing problem cases and by adapting treatment protocols. Annual attendance at key NET-specific educational and research conferences, such as ENETS and CommNETS, is strongly supported, along with participation in specific theranostics conferences and preceptorships. This promotes continuous professional development and the acquisition of new competencies, ensuring that the entire team remains skilled and up to date.

By fostering a strong culture of ongoing learning and collaboration, the service aims to continuously improve patient care and optimize treatment outcomes. 13

**Fig. 4:**
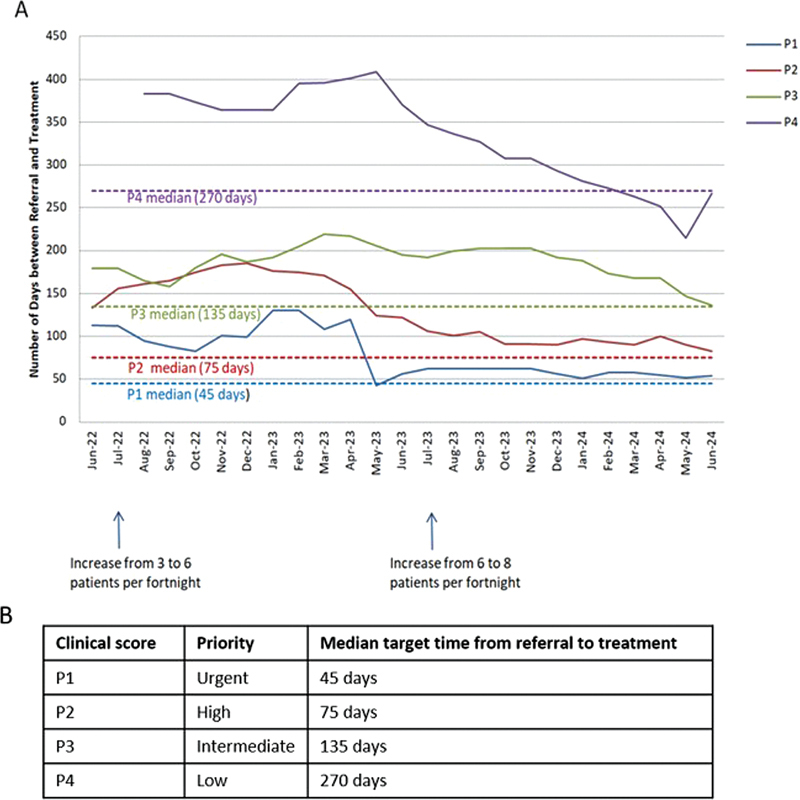
Time between referral and treatment commencement by priority ( **A** ). Clinical priority scoring system ( **B** ).

2. Addressing the “bow wave” and increasing capacity:

The extended period without PRRT access prior to 2019 created a backlog and a surge in referrals that exceeded the estimated 30 patients per year. This “bow wave” saw referral numbers approximately double expectation for the first 4 years, specifically 51 in 2021, 59 in 2022, 64 in 2023, and 22 by June 2024. This puts significant pressure on the PRRT waitlist. The service gradually increased capacity treating three patients per fortnight, expanding to six in July 2022, and then eight in July 2023. In addition, the initial patients had more advanced disease with higher risk of secretory crisis needing support from medical oncology and endocrinology. The “bow wave” is now settling at 4 years after service initiation and patients are now being treated within the ideal time frames ( Fig. 4 ).

3. Treating the right patient at the right time:

A clinical priority scoring system with treatment time frames ( Fig. 4 ) was introduced in 2022 to replace the “first referred, first treated” system. This was done to improve equity and safety of patient care. Treatment time frames also offered a trackable metric for audits, ensuring that patients are prioritized based on clinical risk and treated within a designated time frame, regardless of ethnicity or geographical location.

4. Transitioning from paper to digital:

At the inception of the service, PRRT referrals were submitted via email, printed as hardcopies, and stored in a physical folder in a filing cabinet. These referrals were processed on a “first referred first serve” basis with no digital record of the patient information provided. In April 2022, the implementation of a central digital referral system introduced an electronic patient data capture method process. This transition enabled all patients to have digital summaries of their referral information and allowed for priority placement on an electronic waitlist. The digital summaries are easily accessible throughout the patient's PRRT treatment and are continuously updated to include blood test and imaging results performed during treatment. This has significantly improved the accuracy of tracking and monitoring patients and will be an important data source for our national PRRT database, which is currently being established.

Fig. 4 depicts the “bow wave” and its gradual resolution, achieved by an incremental increase in capacity and working through the backlog of patients at the time-of-service initiation. Accurate waitlist data capture was only possible after implementing digital methods for data management, and so data are shown from June 2022.

5. Increasing administrative support:

Initial insufficient administrative support led to communication breakdowns and inappropriately shifted administrative duties to clinical staff, especially NET Clinical Nurse Specialists (CNSs). Securing full-time administrative support significantly improved communication between the service, referring clinicians, and patients and has enabled NET CNSs to focus on coordinating and delivering clinical care.

6. Social and cultural support:

All patients receive comprehensive support throughout their PRRT journey. Prior to the first specialist assessment (FSA), the team proactively identifies potential barriers to treatment, such as limited social support, low health literacy, financial hardship, or psychological challenges (e.g., anxiety related to scans or treatment). Recognizing that such factors can impact treatment access and adherence, the service works closely with local hospitals and community networks to provide tailored assistance. This includes food and petrol vouchers, transport support, and additional appointments as needed, ensuring patients remain engaged in their care. Strong relationships with local lead health care providers (initiated through the NET MDM) across the country allow the team to optimally utilize local resources and ensure that patients receive individualized and closely coordinated care.

In recognition of the ongoing health inequities experienced by NZ people of Māori ancestry, and with the aim of delivering gold-standard, culturally responsive care, the PRRT service has introduced dedicated cultural support for Māori patients. One of Auckland City Hospital's Kaumātua (respected elder) plays a pivotal role in engaging with Māori patients, providing cultural connection, support, and karakia (prayer) during both the FSA and on the ward before treatment. A kaumātua is an acknowledged leader and guardian of traditional knowledge, genealogy, and cultural customs. Through whakawhanaungatanga—a process of relationship-building based on shared ancestry, friendships, and interests—Māori patients are welcomed into the service in a way that aligns with Te Ao Māori (the Māori worldview). This culturally grounded approach fosters a sense of belonging, trust, and emotional well-being, ensuring Māori patients feel valued, respected, and fully supported throughout their PRRT journey.

## The NZ PRRT Service Today

A recent audit showed that 100 patients completed PRRT treatment (this included completion of induction or maintenance therapy followed by a post treatment dotatate PET/CT at 3 months for response assessment) during the period from June 2021 to July 2024, with a total of 332 PRRT doses administered ( Table 2 ).

**Table 2:** Reason for referral, mode of treatment, burden of disease, tumor type, and functionality of patients treated between June 2021 and July 2024

Component	Number of patients/percentage
Reason for referral	
Disease progression	68
Uncontrolled symptoms	26
Organ compromise	5
Other	1
Mode of treatment	
Induction	83
Maintenance	17
Burden of disease	
Low	13
Moderate	55
High	32
Tumour type	
Well-differentiated NET Grade 1	18
Well-differentiated NET Grade 2	63
Well-differentiated NET Grade 3	4
Paraganglioma	7
Pheochromocytoma	2
Atypical carcinoid	1
Typical carcinoid	3
Histologically indeterminate	1
Other	1
Site	
Stomach	2
Duodenum and ampulla	1
Jejunum and ileum	46
Colon and rectum	1
Pancreas	26
Adrenal gland	1
Thymus	1
Paraganglioma	7
Lung	4
Other	11
Function	
Nonfunctioning	39
Functioning	61

Abbreviation: NET, neuroendocrine tumor.

**Fig. 5:**
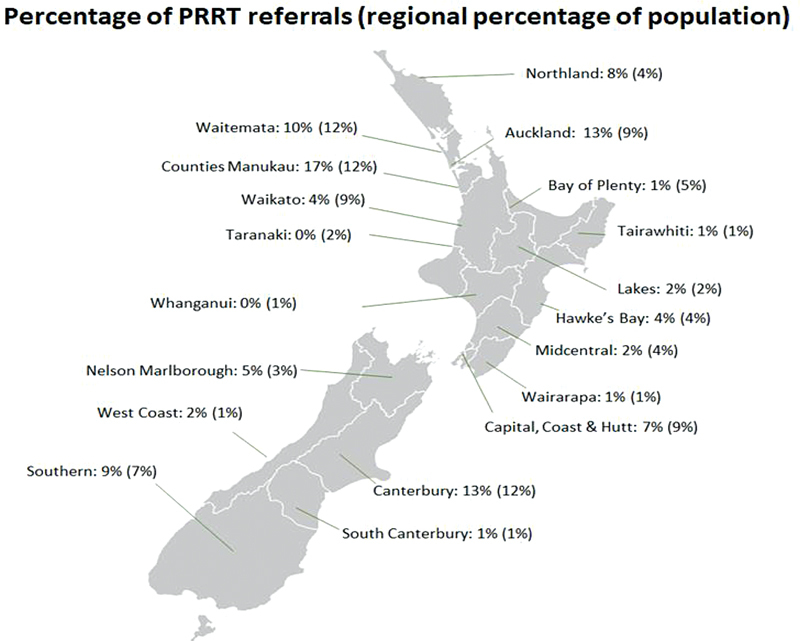
Distribution of patients referred for peptide receptor radionuclide therapy (PRRT) against the general population in New Zealand (NZ). 17

A key aim for this national PRRT service is delivery of care fairly to all people with NETs in NZ. Pleasingly, the regional distribution of the 100 audited patients treated correlates with the regional distribution of NZs general population 17 ( Fig. 5 ).

## Aiming for Continuous Improvement

NZ's PRRT service continues to innovate in collaboration with researchers. An automated prospective national PRRT database is in development with the University of Auckland to evaluate treatment outcomes and safety in real time. Pathways for treatment of NET patients requiring I-131 MIBG therapy have been developed. Collaboration is planned to consider a public radioembolization service. The publicly funded NET MDM and PRRT service collaborates with specialists treating NET patients in private practice to ensure cohesive care irrespective of location. Participation in research and clinical trials will enable the exploration of novel treatments, including new radionuclides and combination therapies, ensuring ongoing innovation in patient care. Regular education and training is provided for clinicians and patients by members of the PRRT service. Continued collaboration with expert centers fosters the value of multidisciplinary care to ensure dissemination of best practices for NET patients throughout NZ.

## Conclusion

NZ's journey to a nationally coordinated, collaborative, and equitable PRRT service is testament to perseverance and the pursuit of equity in health care. Despite significant challenges, the unwavering commitment of clinicians, patient advocates, and the national NET MDM, effective central government leadership, collaboration between public and private health care, and international support has resulted in a robust service aligned with international gold-standard practice. In particular, we acknowledge the drive and courage of people with NETs in NZ who have been critical in delivering this result, and to whom this work is dedicated.
